# AI redefine untargeted metabolomics: estimating chemical amounts for a Human Exposome Project

**DOI:** 10.3389/fpubh.2026.1775284

**Published:** 2026-03-05

**Authors:** Fenna C. M. Sillé, Karolina Kopańska, Carsten Prasse, Thomas Luechtefeld, Thomas Hartung

**Affiliations:** 1 Center for Alternatives to Animal Testing (CAAT), Department of Environmental Health and Engineering, Bloomberg School of Public Health and Whiting School of Engineering, Johns Hopkins University, Baltimore, MD, United States; 2 Department of Environmental Health and Engineering, Bloomberg School of Public Health and Whiting School of Engineering, Johns Hopkins University, Baltimore, MD, United States; 3 Bloomberg School of Public Health, Risk Sciences and Public Policy Institute, Johns Hopkins University, Baltimore, MD, United States; 4 Insilica Inc., Rockville, MD, United States; 5 CAAT-Europe, University of Konstanz, Konstanz, Germany

**Keywords:** artificial intelligence, concentration prediction, exposomics, Human Exposome Project, ionization efficiency, quantitative structure–retention relationships, Threshold of Toxicological Concern (TTC), untargeted metabolomics

## Abstract

The Human Exposome Project aims to map the totality of environmental exposures, but its success relies on transforming qualitative detections into quantitative data. Following our review on AI-driven metabolite identification, this second installment addresses the next critical bottleneck: estimating chemical concentrations in untargeted metabolomics without authentic standards. Translating LC-HRMS signal intensities into absolute concentrations is hindered by the vast variability in ionization efficiency and matrix effects, particularly for xenobiotics where reference standards are unavailable. We review emerging strategies that leverage artificial intelligence—ranging from descriptor-based regression to deep learning on molecular point clouds—to predict ionization response factors. We further evaluate a “matrix-embedded” calibration approach that utilizes ubiquitous endogenous metabolites (e.g., amino acids, lipids) as internal anchors to normalize response scales across studies. These innovations enable “tiered semi-quantification,” allowing the classification of exposures into biologically relevant ranges (e.g., nanomolar vs. micromolar). This stratification facilitates direct integration with toxicological frameworks, such as the Threshold of Toxicological Concern (TTC) and high-throughput bioactivity data (e.g., ToxCast), for rapid risk prioritization. By integrating quantitative AI prediction models with robust quality assurance, untargeted metabolomics can evolve from a qualitative discovery tool into a quantitative engine for exposure science, providing the necessary evidence to link complex chemical exposures to human health outcomes.

## 1 Introduction—From identification to quantification

Mass spectrometry (MS) continues to advance as a foundational technology for the Human Exposome Project, with significant and accelerating improvements in detection sensitivity that enhance its capacity to quantify low-abundance environmental exposures. While MS sensitivity has historically improved at a slower pace than the transistor doubling rate predicted by Moore's Law (approximately 26% annually), recent trends show substantial acceleration—particularly over the past decade—bringing MS improvements close to or even exceeding Moore's benchmark in some applications (1, 2). Industry-reported performance, such as that from SCIEX and Thermo Fisher, has demonstrated nearly a million-fold sensitivity increase over 30 years, with recent instruments like the SCIEX 7500+ and Orbitrap Astral Zoom offering dramatically enhanced signal-to-noise ratios, scan speeds, and multiplexing capabilities (3, 4). Peer-reviewed studies confirm this trajectory: detection limits for compounds like glycine and DDT have improved at rates of 0.2–0.62 on a log scale during 2012–2022, driven by the adoption of high-resolution mass analyzers (e.g., Orbitrap, FT-ICR, MR-TOF) and hybrid systems (1). These gains are underpinned by breakthroughs in ion source design (e.g., nanopore and HES 2.0), mass analyzer resolution, signal preaccumulation, and noise-suppression via advanced spectral averaging (2, 5, 6). Collectively, these developments enable the detection of compounds at femtomolar or even lower concentrations—critical for exposomics, where many xenobiotics occur at trace levels. Importantly, projections suggest that single-molecule detection may be routinely achievable by 2032 (1), establishing MS not only as a pillar of chemical detection but also as an intelligent estimator of internal chemical burden. As AI-enabled pipelines increasingly incorporate MS-derived semi-quantitative data into exposure prediction and risk assessment, these ongoing technological improvements ensure that MS remains central to mapping the invisible chemistry shaping human health.

In the first part of this series, we highlighted how AI has begun to overcome one of the major bottlenecks in untargeted metabolomics—the identification of unknown compounds. By integrating retention-time (RT) prediction, quantitative structure–retention relationships (QSRR), and machine-learning (ML)-based annotation workflows, it is now possible to substantially reduce the number of unidentified spectral features in liquid chromatography–high-resolution MS (LC-HRMS) data (7). Yet even when identities are assigned with high confidence, another fundamental barrier remains: translating instrument signal intensities into quantitative or semi-quantitative concentrations that reflect actual molecular abundances in biological matrices.

This shift from qualitative detection to quantitative inference is critical for exposomics. The ultimate goal of the Human Exposome Project is not merely to catalog the chemicals present in human biospecimens but to estimate the extent of exposure—how much of each compound reaches the internal environment, at what concentrations, and in which individuals or populations. Without this quantitative dimension, the vast majority of detected features remain biologically uninterpretable. Peak intensities alone cannot be compared across studies, related to known toxicological thresholds such as the Threshold of Toxicological Concern (TTC) (8) nor effectively anchored to Adverse Outcome Pathways (AOPs) for safety assessment (9).

Recent exposomics reviews underscore that the limiting step is increasingly not detection, but translation. HRMS can, in principle, support a comprehensive characterization of the internal chemical exposome, yet practical deployment remains constrained by limited analytical versatility, insufficient sensitivity for many ultratrace exogenous chemicals, and incomplete automation of workflows—together motivating stronger harmonization and shared infrastructure (10). In parallel, suspect and non-target screening is increasingly viewed as an early-warning tool for identifying chemicals of emerging concern in human biomonitoring and supporting risk assessment, but its regulatory traction is hindered by uneven methodological harmonization, reference resources, and reproducible computational pipelines (11). These assessments converge on a common need: methods that can convert HRMS signal into defensible, comparable exposure estimates without requiring standards for every feature.

Absolute quantification in untargeted metabolomics remains challenging because ionization efficiency, matrix effects, and instrument-specific response factors vary over several orders of magnitude and are rarely known for compounds lacking authentic standards. The result is a landscape rich in chemical discovery but poor in exposure precision. Bridging this gap requires new strategies that use data-driven calibration, AI-based response modeling, and endogenous reference metabolites to infer concentrations directly from untargeted datasets.

As retention-time prediction and probabilistic annotation mature, the field is poised to tackle this next frontier—quantification without standards. Achieving even approximate, tiered estimates of concentrations (e.g., < 1 nM, 1–10 nM, 10–100 nM, >1 μM) will transform untargeted metabolomics into a semi-quantitative engine for exposure science, enabling prioritization of xenobiotics by magnitude of occurrence and alignment with health-relevant benchmarks. In this second installment, we outline how metabolomics can evolve from identifying “what is there” to estimating “how much is there,” thereby turning detection into inference and data into evidence for environmental health decision-making.

## 2 The challenge of ionization efficiency

The transition from identifying a molecular feature to estimating its abundance is fundamentally limited by the physics and chemistry of ionization efficiency—the probability that a molecule entering the ion source of an MS is converted into a detectable ion. In electrospray ionization (ESI), the dominant interface in LC-HRMS-based metabolomics, this process depends on a complex interplay of physicochemical parameters including molecular polarity, surface activity, pKa, solvation energy, and the charge competition among co-eluting species. Even under carefully controlled conditions, response factors can vary by several orders of magnitude between compounds of similar mass but different structure. Consequently, signal intensity cannot be assumed to scale linearly with concentration across chemical classes.

### 2.1 Scope and application domain

**Figure 1:**
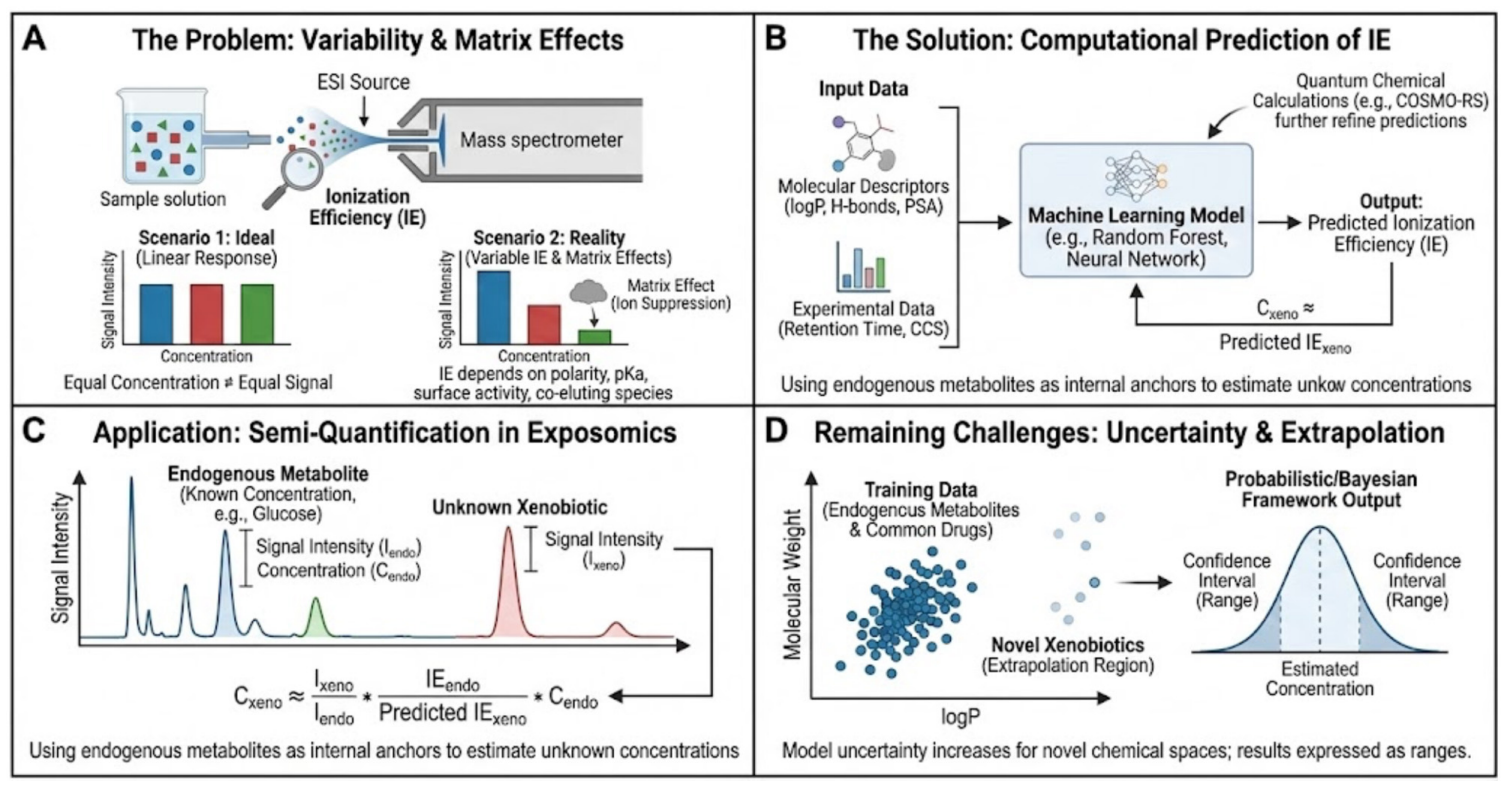
The challenge of ionization efficiency in untargeted metabolomics and exposomics and emerging computational solutions. **(A)** The fundamental problem in electrospray ionization (ESI) is that mass spectrometry signal intensity does not scale linearly with analyte concentration. Response varies significantly due to inherent physicochemical properties (e.g., polarity, surface activity) and matrix effects such as ion suppression by co-eluting species. **(B)** To address this, computational methods use molecular descriptors (e.g., logP, polar surface area), experimental data (retention time, CCS), and quantum chemical calculations to train machine learning models that predict ionization efficiency (IE). **(C)** These predicted IE values enable semi-quantification in exposomics. Unknown xenobiotics can be quantified relative to ubiquitous endogenous metabolites with known concentrations (internal anchors) by correcting for the difference in their predicted ionization response. **(D)** A major remaining challenge is model extrapolation to novel xenobiotics that occupy chemical spaces far from the training data. To account for this increased uncertainty, probabilistic or Bayesian frameworks are used to express concentration estimates as confidence intervals rather than single point values.

This semi-quantification framework applies to three distinct use cases with varying confidence levels (Figure 1):

Primary scope—Annotated compounds without standards: Features with confident structural assignments (Level 2 annotation per Schymanski et al.) but lacking authentic standards. Expected accuracy: 3–10 fold error with defined confidence intervals.

Secondary scope—Partially characterized features: Features with molecular formula or partial structural information (Level 3–4 annotation). Expected accuracy: 10–50 fold error, suitable for order-of-magnitude binning.

Exploratory scope—Unannotated features: Complete unknowns with only m/z and retention time. Expected accuracy: 50–100 fold error, applicable only for extreme concentration flagging (e.g., >100 μM).

The framework's reliability decreases from primary to exploratory scope, and uncertainty estimates must be adjusted accordingly. For regulatory or clinical applications, only primary scope estimates should be considered actionable.

### 2.2 Ionization variability: the core analytical challenge

This variability has long been recognized as a major obstacle to absolute quantification in untargeted workflows. Unlike targeted assays, where each analyte is measured against an authentic standard or an isotopically labeled analog, untargeted metabolomics detects thousands of features for which no calibration curves exist. Matrix effects further complicate interpretation: ion suppression or enhancement by co-eluting constituents leads to sample-specific response biases that defy simple normalization. As a result, most untargeted studies report relative abundances or fold-changes rather than concentrations—useful for pattern discovery but inadequate for exposure assessment.

Recent advances, however, suggest that ionization efficiency follows systematic and predictable patterns that can be learned computationally. Experimental studies using reference compounds across chemical space have shown that response factors correlate with descriptors such as logP, hydrogen-bond donor and acceptor count, polar surface area, and adduct type (12, 13). ML models trained on these descriptors, sometimes augmented with chromatographic retention time or ion-mobility collision cross-section, can predict ionization efficiency within a few-fold accuracy. Recently, approaches integrating quantum chemical calculations such as COSMO-RS have further refined these predictions (14). Such methods have been successfully applied to estimate serum concentrations of complex analytes like hydroxylated polychlorinated biphenyls, demonstrating their utility for real-world biological matrices (15). Such models transform a purely empirical nuisance parameter into a quantifiable property, enabling estimation of response factors even for uncharacterized compounds. A challenge with using these approaches is communicating the confidence of the prediction. A lot of times a point estimate is given with no confidence interval (16). This is stressed by bounded response factor method, where the accuracy, uncertainty, and reliability of the measurements are defined and reported and every prediction has a confidence interval.

For exposomics, this capability is transformative. If the ionization behavior of unknowns can be inferred from their structural or physicochemical similarity to compounds with known concentrations—particularly endogenous metabolites with well-established clinical ranges—then LC-HRMS signals can be converted into semi-quantitative concentration estimates. This approach effectively uses the biological matrix itself as an internal calibration environment, turning ubiquitous metabolites such as glucose, creatinine, or tryptophan into built-in reference anchors for response modeling.

Nevertheless, major challenges remain. The relationship between chemical structure and response is non-linear and context-dependent; matrix composition, solvent additives, and instrumental tuning can all modulate ionization dynamics. Moreover, xenobiotics often occupy sparsely sampled regions of chemical space, where model extrapolation is uncertain. To address these issues, probabilistic frameworks that output prediction confidence intervals are being explored, allowing concentration estimates to be expressed as ranges rather than point values.

Understanding and modeling ionization efficiency is thus the critical first step toward quantitative untargeted metabolomics. It provides the theoretical and computational foundation for the next sections—leveraging known clinical metabolites as internal calibrants and developing AI-driven models to translate intensity into approximate concentration, moving exposomics closer to quantifying the real-world chemical exposures that shape human health.

## 3 Using known clinical metabolites as internal calibrants

The pervasive presence of well-characterized endogenous metabolites in human biofluids provides a powerful yet underexploited opportunity to transform untargeted LC-HRMS from a purely qualitative to a semi-quantitative science. Human plasma and serum contain hundreds of metabolites with reliably reported concentration ranges—glucose, lactate, amino acids, urea, creatinine, cholesterol, and numerous others—measured across decades of clinical chemistry and metabolomics studies. These molecules appear consistently in virtually all untargeted datasets and thus can serve as in-matrix reference points for calibrating ionization response.

The rationale is straightforward: if the LC-HRMS peak intensities of compounds with known physiological concentrations are recorded under the same analytical conditions as xenobiotics of interest, the relationship between signal intensity and true abundance can be empirically modeled. This approach converts the metabolome itself into a self-referential calibration system, bypassing the need for extensive external standardization. Conceptually, each identified endogenous metabolite acts as an internal anchor linking measured ion currents to expected molar quantities; the collective set of anchors defines a response surface that can be interpolated for unknown compounds.

To implement this, one first compiles a panel of in the order of 20–50 confidently identified metabolites spanning a wide range of physicochemical properties—polar (e.g., glucose, serine), amphipathic (e.g., tryptophan, phosphocholine), and hydrophobic species (e.g., palmitate, cholesterol). Their literature-derived concentration ranges (for plasma typically 10 nM−10 mM) are used as approximate quantitative references. For each metabolite, the observed peak area (normalized for sample volume and injection load) is paired with descriptors such as retention time, predicted logP, charge state, and adduct type. These data serve as training inputs for regression ML models—ranging from random forests and gradient boosting to deep neural networks or Bayesian inference frameworks—that learn the relationship between molecular features and ionization efficiency.

Once trained, the model predicts relative response factors for all detected compounds. By combining these predictions with measured peak intensities, one obtains semi-quantitative concentration estimates for both annotated and unannotated features, including xenobiotics. The method is most robust for analytes whose structural or physicochemical attributes fall within the calibration chemical space; extrapolation beyond this domain should be accompanied by uncertainty estimates or tiered concentration ranges (e.g., < 1 nM, 1–10 nM, 10–100 nM, >1 μM); uncertainty estimates should be included for both chemicals within the chemical space and those that are outside (having a higher uncertainty).

A key advantage of this endogenous-calibrant strategy is its matrix fidelity, utilizing principles analogous to Best-Matched Internal Standard (BMIS) normalization (17). Because the calibration compounds are measured in the same biological matrix, they experience identical ion-suppression, extraction, and chromatographic effects as the unknowns, minimizing systematic bias. Moreover, this approach is inherently scalable to large exposomic datasets and retrospective analyses: it does not require re-analysis of samples or the addition of external standards. In contrast to traditional absolute quantification, which demands compound-specific calibration curves, this framework yields an approximate but consistent abundance scale applicable across thousands of analytes.

Several proof-of-concept studies support the feasibility of this idea. Liigand et al. (12) demonstrated that ionization-efficiency models trained on structurally diverse compounds could predict response factors for unmeasured chemicals within a 5- to 10-fold accuracy window. Beyond single-lab feasibility, Wang et al. (18) demonstrated that response-factor prediction can be made transferable across laboratories by calibrating a universal ionization-efficiency model to a specific instrument using a limited set of “transformation compounds.” In a two-laboratory study (triple quadrupole vs. QTOF) covering 134 pesticides spiked into six cereal matrices, the resulting standard-free concentration estimates were strongly concordant between laboratories (*R*^2^ ≈ 0.85; slope ≈ 0.95), with average deviations on the order of only a few-fold. This type of cross-lab quantitative harmonization is exactly what exposomics needs to move from intensity lists to comparable exposure metrics that can be interpreted against biological activity thresholds and used for prioritization when authentic standards are unavailable. Palm and Kruve (13) extended this to deep-learning architectures capable of absolute quantification of unidentified features in non-targeted LC/HRMS: they developed a quantification approach, where LC/ESI/HRMS descriptors are used for quantification of compounds even if the structure is unknown. Go et al. (19) and later Ferro et al. (20) further highlighted the potential of reference-standardization approaches that harmonize intensity scales across laboratories, making concentration comparisons between studies realistic even without authentic standards.

In exposomics, such semi-quantitative scaling provides precisely the information required for prioritization. By estimating whether a detected xenobiotic is likely present at picomolar, nanomolar, or micromolar levels, researchers can immediately place exposures in the context of biological activity thresholds [e.g., ToxCast (63) AC_50_ distributions, TTC values]. This moves the field from descriptive lists of “molecular features” toward actionable exposure metrics. Importantly, even coarse-grained estimates can dramatically enhance interpretation in population studies, supporting dose-response modeling and probabilistic risk assessment when absolute quantification is unfeasible. This also allows for prioritization of compounds (e.g., due to their concentrations being at or above the toxicity threshold.

However, many xenobiotics in exposomics (e.g., halogenated persistent chemicals, surfactants, polymer additives) occupy sparsely sampled or divergent chemical spaces that may not be adequately represented by the chosen anchors. We will need to define or test an explicit applicability-domain check to prevent extrapolation from the anchor set to xenobiotic classes with divergent ionization behavior in line with established OECD validation principles requiring applicability domain definition for reliable QSAR predictions. This concern is validated by research showing that ionization behavior varies significantly across chemical classes (21, 22).

**Figure 2:**
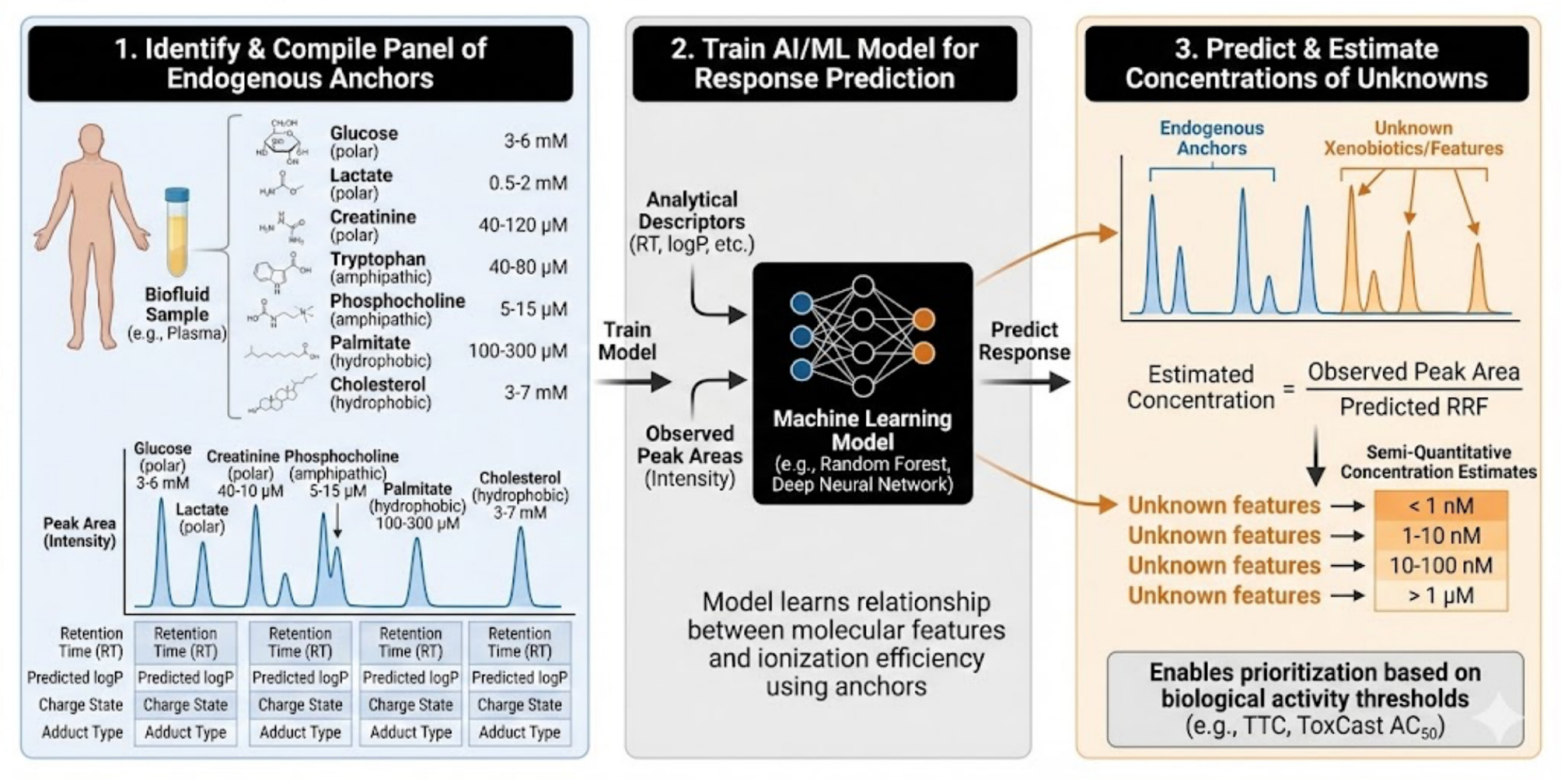
Using known clinical metabolites as internal calibrants for semi-quantification in untargeted metabolomics. This three-step workflow illustrates how endogenous metabolites with well-characterized concentration ranges can serve as in-matrix anchors to estimate the abundance of unknown features. (1) Identify and Compile Panel of Endogenous Anchors: A diverse set of ubiquitous metabolites (e.g., polar glucose, amphipathic tryptophan, hydrophobic cholesterol) is identified in a biofluid sample (e.g., plasma). Their literature-derived clinical concentration ranges and analytical descriptors (retention time, predicted logP, etc.) are compiled along with their observed peak areas. (2) Train AI/ML Model for Response Prediction: These analytical descriptors and observed peak intensities are used to train a machine learning model (e.g., random forest, deep neural network) to learn the relationship between molecular features and ionization efficiency. (3) Predict and Estimate Concentrations of Unknowns: The trained model predicts the Relative Response Factor (RRF) for unknown xenobiotics based on their analytical descriptors. The concentration of these unknowns is then estimated by dividing their observed peak area by their predicted RRF. The final output provides semi-quantitative estimates in tiered ranges (e.g., nanomolar vs. micromolar), enabling the prioritization of features based on biological activity thresholds (e.g., TTC, ToxCast AC_50_).

In summary, leveraging known clinical metabolites as internal calibrants offers a pragmatic route to standard-free quantification in untargeted metabolomics (Figure 2). It aligns analytical chemistry with the wealth of physiological data accumulated through clinical biochemistry, effectively turning the human metabolome into its own quantitative ruler. The following section explores how this concept can be generalized and automated through AI-driven frameworks that integrate molecular descriptors, retention behavior, and predicted ionization efficiency into unified models for concentration estimation across the exposome.

## 4 Machine-learning frameworks for semi-quantification

The emergence of AI and ML methods has transformed how we interpret MS data, enabling not only improved identification—as discussed in Part I (7)—but also a new paradigm of quantitative inference without standards. Comprehensive strategies for drawing such quantitative conclusions have been outlined by Kruve (23), establishing the theoretical basis for converting non-targeted data into actionable evidence. Rather than relying on compound-specific calibration curves, ML models can learn statistical relationships between molecular structure, ionization efficiency, chromatographic behavior, and observed signal intensity. These data-driven mappings allow estimation of approximate concentrations even for uncharacterized analytes, thereby extending the reach of quantitative metabolomics into the vast chemical space of the exposome.

Early efforts in this direction relied on molecular-descriptor-based regression models. Random forest and support-vector regression approaches, trained on sets of authentic standards spanning diverse chemical classes, used descriptors such as logP, polar surface area, pKa, charge, and retention time as inputs to predict ionization efficiency or relative response factors. Liigand et al. (12) demonstrated that such models could estimate analyte responses within a median 5-fold error in electrospray LC/MS. Building on this, Abrahamsson et al. (24) validated ML approaches for *in silico* quantification across broad chemical lists without analytical standards. More recently, Abrahamsson et al. (25) refined this by modeling Relative Response Factors (RRF) for small molecules, offering improved precision for electrospray ionization. Palm and Kruve (13) further advanced the field by showing that deep neural networks further reduced the prediction error range, approaching 2- to 3-fold accuracy for compounds within the calibration domain. They demonstrated a complementary structure-free approach to absolute quantification in non-targeted LC/HRMS by predicting response factors directly from LC/MS descriptors (including polarity- and pH-dependent intensities, retention-time descriptors, m/z, and detectability/adduct-related features) using regularized random-forest models. Across six models (three mobile-phase pH values × two ionization modes), the hold-out test set showed mean errors of ~10-fold for both response-factor and concentration prediction, while blind-sample validation in ultrapure and tap water achieved mean errors of ~6-fold with ~88–90% of compounds within 10-fold. Notably, these LC/MS-descriptor models substantially outperformed common semi-quantitative shortcuts such as the equal-response assumption and closest-eluting-standard assignment, which can yield very large errors. These accuracies, while likely insufficient for regulatory quantification, reflect heterogeneous datasets; standardized benchmarks are required for direct cross-approach comparison.

Recent advances incorporate multi-modal information. Retention time, predicted from QSRR or RT-Transformer models, has emerged as a valuable proxy for physicochemical behavior influencing ionization. Including RT as a feature in response-factor prediction markedly improves performance, capturing co-variations between hydrophobicity, elution order, and signal response (7). Other frameworks integrate fragmentation intensity patterns, ion-mobility collision cross-section, and adduct type, yielding richer descriptors of molecular detectability. Deep learning architectures trained on thousands of annotated spectra—ranging from graph neural networks to point cloud-based models like 3D-MSNet (2)—can automatically learn latent representations of structure that generalize across chemical families and instruments.

Recent work underscores that “response” in LC–HRMS is not dictated by molecular ionization efficiency alone, but is strongly shaped by experimental context—an insight that helps refine how AI-enabled semi-quantification should be operationalized for exposomics. In particular, Chu et al. (26) introduced a statistically driven design-of-experiments (DoE) framework to model response factors as an explicit function of chromatographic and sampling variables (e.g., flow rate, sample loading, ESI voltage, pH, organic phase, and co-elution), demonstrating that these parameters can contribute as much as—or more than—structure-derived ionization-efficiency predictors. Their bootstrapped response factor approach achieved an approximately 10 fold improvement in reference-free concentration estimates compared with ionization-efficiency–centric baselines, and importantly enabled accurate, sample-specific quantification even without compound identity—an attractive property for the “chemical dark matter” typical of untargeted exposomics (26). Together with ionization-efficiency prediction (12, 13) and matrix-embedded calibration using ubiquitous endogenous metabolites as internal anchors (7), these results argue for next-generation pipelines that are both chemistry-aware and method-aware: leveraging endogenous anchors to stabilize intensity scales while incorporating standardized experimental metadata and uncertainty-aware response-factor models to improve transferability and reproducibility across laboratories. A comparative summary of current response factor modeling approaches, including ionization-efficiency-based, endogenous-calibrant, hybrid ML, DoE-based models and bounded response factors, is provided in Table 1. Given the absence of community benchmarks, we synthesized published error ranges from ESI LC–HRMS studies to provide a pragmatic overview. The ranges compiled in Table 1 derive from heterogeneous datasets and protocols; direct cross-approach comparisons require standardized benchmarks and shared test sets.

**Table 1:** Comparative overview of response factor modeling methods for reference-free quantification in LC-HRMS.

**Approach**	**Key references**	**Input features**	**Experimental info required**	**Need for compound identity**	**Quantification accuracy (fold error)**	**Scalability to unknowns**	**Strengths**	**Limitations**
Ionization Efficiency–Based (IE-only)	(12, 13)	Molecular descriptors (e.g., logP, PSA, H-bonding), optionally retention time	No	Yes	~5–10X	Limited outside chemical space of training set	High throughput; applicable to large annotated datasets	Requires accurate structure; assumes ionization dominates response
Endogenous Metabolite Scaling	(7) (this manuscript)	Peak intensities of internal calibrants + molecular features of unknowns	No (uses in-matrix anchors)	No (if binned)/Yes (for refined scaling)	~3–10X (depending on anchor quality)	Moderate to high	Matrix-matched calibration; harmonizes across studies	Depends on quality and range of anchor metabolite coverage
ML-Based Hybrid (IE + RT + Adduct)	(2, 13)	Molecular descriptors, RT/QSRR, adduct type, CCS (if available)	No	Yes	~2–5X	Moderate (depends on feature annotation)	Improved generalization via multi-modal features	Requires accurate annotation; performance limited by training domain
Statistical DoE–Driven Response Factor model	(26) (PNNL-36690)	Flow rate, sample loading, ESI voltage, pH, solvent, co-elution %, optionally logIE	Yes (DoE-based parameterization)	No (sample-specific model); Yes (compound-specific variant)	~0.5X (sample-specific) ~0.7X (compound-specific)	High (including unannotated features)	10X improved accuracy; instrument-agnostic if reparameterized; open-source workflows	Requires experimental design and treatment space coverage; current models trained on nanoLC only
Bounded Response Factor (RF) method	(16)	Training RF distribution (RF = intensity/concentration); bootstrap RF quantiles (e.g., RF0.025, RF0.975)	Yes (requires RF distribution from spiked/known-concentration training set)	No	~130–150X (upper confidence limit error; ENTACT)	High (including unannotated features)	Provides conservative concentration bounds with confidence limits; no structure needed	Wide intervals; accuracy depends on representativeness of RF distribution and instrument/matrix effects

Interpretation: The fold-error ranges presented below are compiled from heterogeneous studies using different chemical sets, matrices, instruments, and evaluation metrics. These values should NOT be interpreted as direct performance comparisons between methods. Standardized benchmarks using common test sets are urgently needed for meaningful cross-method evaluation. The ranges provided serve only to illustrate the general performance envelope of current approaches.

Bayesian and probabilistic learning adds another dimension by quantifying uncertainty. Instead of providing a single estimated concentration, these models output posterior distributions or confidence intervals, allowing analysts to propagate uncertainty into downstream exposure or risk calculations. This is particularly important for xenobiotics outside the calibration chemical space, where predictions may be less reliable. Probabilistic models also enable hierarchical learning, combining global priors derived from large public datasets with local calibration using endogenous metabolites, effectively adapting to laboratory-specific conditions while retaining generalization.

In practical terms, the semi-quantification pipeline proceeds as follows:

1. Detect features and annotate known endogenous calibrants (Section 3).
2. Compute or import molecular descriptors, predicted retention times, and ionization parameters for all detected compounds.
3. Train or apply an ML model to estimate response factors (ionization efficiencies).
4. Convert normalized peak areas to concentration estimates, expressing results in log-scale or tiered bins (e.g., < 1 nM, 1–10 nM, 10–100 nM, >1 μM).
5. Rank chemicals or features by estimated concentration and cross-reference with toxicological thresholds such as ToxCast AC_50_ or TTC benchmarks.

The performance of these frameworks is typically evaluated by fold-error metrics rather than absolute bias, reflecting the goal of approximate magnitude estimation rather than exact quantification. For many exposomic applications, achieving concentration accuracy within one order of magnitude is sufficient to distinguish background from biologically relevant exposures. When combined with reproducible QA/QC procedures and transparent uncertainty reporting, ML-based semi-quantification represents a pragmatic compromise between feasibility and interpretability.

Several software environments already implement these principles. Tools such as *MetaQuant* (64), *B-MIS* (17), and the *Retip* (65) framework allow users to couple ionization-efficiency prediction with metabolite annotation, automating the conversion of LC-HRMS intensities into exposure-relevant quantities. Integration into FAIR-compliant (66) workflows and public repositories [e.g., MetaboLights (68), METLIN (67)] ensures that training data continue to expand, enhancing model robustness and transferability across laboratories.

In summary, ML-based estimation of ionization efficiency and response factors bridges the long-standing gap between identification and quantification in untargeted metabolomics. By transforming complex instrument responses into approximate molecular concentrations, these models provide the quantitative scaffolding required to link exposomic data with toxicological evidence. The next step is to operationalize these estimates within tiered exposure assessment frameworks, connecting measured abundance to biological relevance—a subject explored in the following section.

## 5 From intensities to exposure tiers

The ultimate goal of moving from identification to quantification in untargeted metabolomics is to place detected compounds within an exposure–response context. For exposomics, this means translating LC-HRMS signal intensities—once considered relative and qualitative—into approximate concentration tiers that reflect biologically and toxicologically meaningful ranges. Such semi-quantitative stratification provides a bridge between analytical chemistry and environmental health science, allowing researchers to assess whether an exposure is likely trivial, background-level, or potentially of concern.

### 5.1 Tiered semi-quantification as a pragmatic framework

Absolute quantification for thousands of features is infeasible: authentic standards exist for only a small fraction of detected chemicals, and inter-laboratory differences in ionization response prevent universal calibration. Tiered estimation offers a tractable compromise. consistent with emerging strategies for uncertainty estimation in non-targeted analysis (16, 27, 28). Rather than aiming for single-value concentrations, features are binned into logarithmic or categorical ranges—commonly < 1 nM, 1–10 nM, 10–100 nM, > 1 μM—based on modeled response factors and normalized signal intensities. These tiers align with orders of magnitude of exposure relevant to human biology and toxicology: the nanomolar domain for endogenous signaling molecules, the micromolar domain for nutritional or pharmacological compounds, and picomolar or lower for many xenobiotic contaminants.

Such coarse quantification is not a limitation but an enabler as it allows immediate contextualization of detections relative to reference frameworks such as the Threshold of Toxicological Concern (TTC): To apply the TTC concept, estimated plasma or serum concentrations derived from non-targeted analysis, and binned into concentration ranges, can be translated to human exposure doses using physiologically based pharmacokinetic (PBPK) models. Outputs of these models, along with the structural information about the detected compounds, can then be compared to TTC-based human relevant doses, allowing for early risk assessment decisions without requiring precise quantification. Similarly, a chemical estimated to occur at micromolar levels with a ToxCast AC50 of 0.5 μM warrants higher prioritization than one in the picomolar range with an AC_50_ of 100 μM, even if both lack precise quantification. Alternatively, QSAR frameworks in ecotoxicology such as ECOSAR (59) or more general toxicology such as T.E.S.T (29) might be useful too. In population studies, tiered concentration data also facilitate semi-quantitative exposure–response modeling, enabling dose-binning or probabilistic regression approaches without overstating numerical precision.

### 5.2 Integrating quantitative tiers with biological inference

The conversion of peak intensity to concentration tier involves several normalization steps—signal correction for batch effects, model-based adjustment for ionization efficiency (Section 4), and, where possible, calibration using endogenous metabolites (Section 3). The resulting abundance tiers can then be coupled with biological readouts such as transcriptomics, proteomics, or health outcomes to support causal inference. This integrated strategy transforms the metabolome from a descriptive snapshot into an analytically scaled map of internal exposures.

In exposome-wide association studies (ExWAS), for example, semi-quantitative tiers enable weighting of features by estimated internal dose. This allows prioritization not only by statistical association strength but also by plausible biological potency. Likewise, incorporating predicted concentrations into toxicokinetic models supports back-calculation of external exposure doses or estimation of bioaccumulation factors, strengthening translational relevance for risk assessment.

### 5.3 Advantages for reproducibility and harmonization

Another key advantage of tiered quantification is comparability. Because the categories are defined by relative orders of magnitude rather than absolute intensities, they can be reproduced across instruments, laboratories, and studies. The approach naturally tolerates systematic offsets in detector response, focusing instead on relative abundance scaling. Harmonization efforts—such as the NORMAN network (69) for environmental non-target screening and the Metabolomics Quality Assurance & Quality Control Consortium (MQACC) (70)—are now advocating tier-based reporting as a practical path toward inter-laboratory data interoperability. This philosophy aligns with the FAIR principles, emphasizing transparency and transferability over nominal accuracy. Empirical benchmarking highlights why harmonization must go beyond “intensity-as-amount” heuristics. In a preparatory study for the NORMAN semi-quantification interlaboratory comparison, four surrogate strategies were evaluated and the “closest-eluting standard” method—although uniquely applicable when only a feature is available—showed the poorest performance (up to 2000-fold error), while no single approach performed reliably across analytes (30, 31, 60). This supports tiered reporting and motivates parallel, model-informed approaches (e.g., ionization-efficiency prediction), including explicit handling of common adduct chemistry (e.g., Na^+^/${\text{NH}}_{4}^{+}$) to improve robustness across chemical classes and laboratories (57).

### 5.4 Toward quantitative exposomics

Ultimately, the ability to assign approximate concentration tiers converts metabolomics from a tool of chemical discovery into one of exposure science. It allows integration with toxicological and epidemiological frameworks, supports probabilistic risk ranking, and provides a first-order approximation of internal dose for thousands of chemicals simultaneously. When combined with predicted bioactivity thresholds, kinetic modeling, and pathway mapping, these estimates can inform regulatory prioritization, surveillance, and mechanistic hypothesis generation.

However, the proposed tier boundaries (less than 1 nM, 1–10 nM, 10–100 nM, greater than 1 uM) are not yet empirically calibrated against median bioactivity concentrations (AC_50_) or TTC values across real datasets, and no misclassification or uncertainty metrics are reported. Without quantitative benchmarking, the alignment between tiers and biological potency remains unverified, risking misprioritization. We thus have to note that tiers are designed for screening-level decisions. For many exposomic applications, achieving concentration accuracy within one order of magnitude is sufficient to distinguish background from biologically relevant exposures.

**Figure 3:**
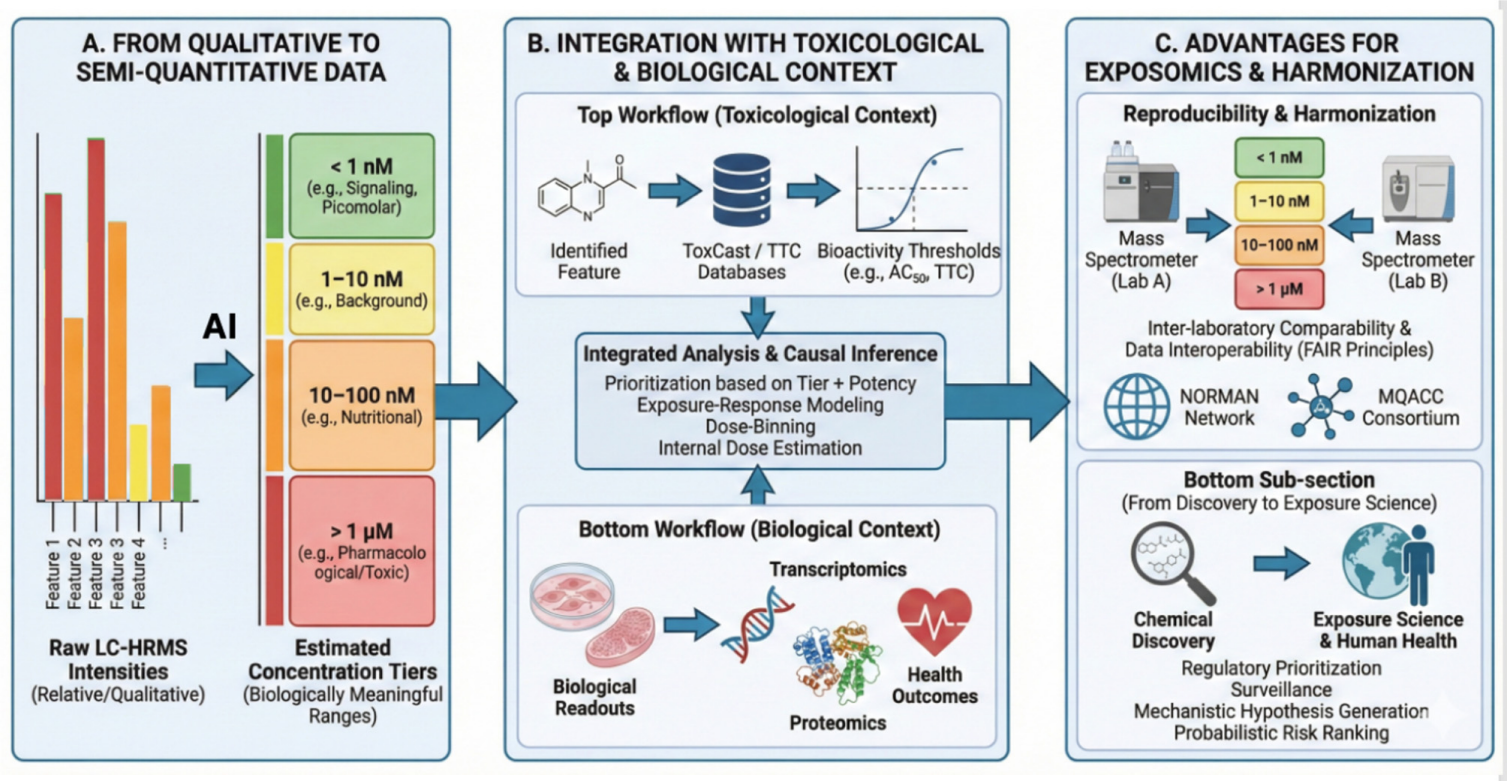
Workflow for transforming raw intensities into meaningful exposure tiers in untargeted metabolomics and exposomics. This figure illustrates the multi-step process of converting qualitative data into actionable insights. **(A)** The conversion of raw, relative LC-HRMS signal intensities into semi-quantitative concentration tiers based on biologically relevant ranges (e.g., signaling, nutritional, pharmacological). **(B)** The integration of these estimated concentration tiers with toxicological data (e.g., ToxCast databases, bioactivity thresholds), biological readouts (e.g., transcriptomics, proteomics, health outcomes), ECOSAR or T.E.S.T to facilitate integrated analysis, causal inference, and prioritization. **(C)** The advantages of this tiered approach, including enhanced reproducibility and harmonization across different laboratories and instruments (supporting FAIR principles), and the transition from mere chemical discovery to comprehensive exposure science for regulatory and risk assessment purposes.

In this sense, semi-quantitative metabolomics (Figure 3) serves as the analytical backbone of the Human Exposome Project, turning detection into inference and enabling data-driven decisions about which exposures matter most. As quantification models mature, the tiers themselves can be refined into continuous probabilistic concentration distributions, providing increasingly precise—but still transparent—representations of uncertainty. Box 1 suggests a minimal reporting framework.

## 6 Integrating quantification into AI-driven exposomics pipelines

The convergence of HRMS, ML, and multi-omics integration is transforming untargeted metabolomics into a data-intensive, inference-driven science. While Part I (7) outlined how AI enhances compound identification through retention-time prediction and probabilistic annotation, the next step is to embed quantitative inference into the same analytical ecosystem. By coupling identification and quantification within unified computational frameworks, metabolomics can evolve from generating feature lists to delivering exposure metrics directly interpretable for toxicology and public health.

### 6.1 From analytical signal to probabilistic quantity

An AI-enabled quantification pipeline begins with feature detection and annotation, followed by automated estimation of response factors and concentration tiers. Deep-learning models trained on large-scale annotated datasets—such as METLIN, HMDB, or METASPACE (71)—can simultaneously predict retention time, ionization efficiency, and even fragmentation intensities from molecular structure. These predictions form the basis of multi-parameter scoring, where each candidate compound receives integrated probabilities for identification and abundance. In practice, the workflow begins with advanced feature extraction, employing tools like JPA (32) to maximize chemical coverage, followed by ML-based cleaning—such as MetaClean (33)—to rigorously filter false-positives.

1. Identification engine (as in Part I): mass- and RT-based candidate ranking using tools such as *SIRIUS* (74), *MetFrag* (73), or *KGMN* (72);
2. Quantification engine: ML-based prediction of ionization efficiency using descriptors, RT, and matrix context;
3. Calibration layer: Bayesian correction using endogenous metabolite anchors, generating posterior distributions of estimated concentrations.

Together these components transform a raw intensity matrix into a probabilistic concentration landscape—a representation of what compounds are present, how confidently they are identified, and at what approximate levels.

### 6.2 Integrating across modalities and scales

Quantitative inference gains robustness when it is integrated across complementary analytical and biological dimensions. Coupling metabolomics with transcriptomics, proteomics, and exposomic metadata enables causal interpretation: changes in metabolite abundance tiers can be related to gene-regulatory or protein-interaction networks affected by specific exposures. Furthermore, linking modeled concentrations to spatial or temporal data (e.g., geolocation, personal sensors, or time-resolved sampling) yields the dynamic perspective needed for exposure trajectory analysis.

Box 1 **Minimal reporting framework for semi-quantitative metabolomics.**

**Required metadata:**

- Instrument: Model, ionization mode (ESI+/ESI-), mass analyzer type
- LC conditions: Column type, gradient profile, flow rate, mobile phases
- Adduct handling: Expected adducts ([M+H]+, [M+Na]+, etc.), deconvolution approach
- Matrix type: Plasma, serum, urine, tissue (specify preparation method)

**Quality control elements:**

- Pooled QC: Injection frequency (recommend every 10 samples), %CV thresholds
- Drift correction: Method used (LOESS, linear, none), correction metrics
- Batch effects: Normalization approach, between-batch bridging samples
- Detection limits: Method LOD/LOQ estimation for tier boundaries

**Uncertainty reporting:**

- Chemical space: Applicability domain definition (Tanimoto similarity threshold)
- Confidence tiers: Probability ranges for each concentration bin
- Anchor coverage: Number and chemical diversity of endogenous calibrants
- Model metrics: Cross-validation error, test set performance if available

**Data sharing requirements:**

- Raw files: Repository (e.g., MetaboLights, MassIVE) with accession number
- Processed data: Peak table with original and corrected intensities
- Model parameters: Ionization efficiency model weights/code
- Calibration metadata: Endogenous anchor concentrations and sources

AI frameworks are uniquely suited for such multi-modal fusion. Transformer architectures and graph-based learning can align heterogeneous inputs—spectral, chemical, biological, and contextual—into shared latent spaces, allowing discovery of exposure–response relationships that would remain hidden in univariate analyses. These models can also integrate toxicological priors, for instance by weighting predicted concentrations by known bioactivity thresholds or physicochemical persistence parameters, producing an exposure-risk score for each detected feature.

### 6.3 Toward automated, FAIR, and transparent workflows

The full potential of AI-driven quantification lies in automation and reproducibility. Implementing standardized, open-source pipelines ensures that the transformation from raw data to quantitative inference is transparent and repeatable. As emphasized in recent perspectives, AI/ML is already central to untargeted metabolomics/exposomics for peak alignment, feature selection, and annotation of unknowns—yet MS1 feature intensities still typically function as semi-quantitative inputs rather than interoperable exposure metrics (34). Embedding calibration metadata (anchors, response-factor priors, uncertainty) alongside spectra in FAIR repositories therefore becomes as important as sharing the raw data, because it enables models to be retrained and quantification performance to be benchmarked transparently across laboratories. FAIR-compliant infrastructures—such as the *MetaboLights, GNPS/MassIVE* (75), and *NORMAN Suspect List Exchange* ecosystems—can host not only raw spectra but also model predictions, calibration metadata, and uncertainty metrics. This openness allows models to be continuously retrained and benchmarked across laboratories, gradually improving performance and generalizability.

Integration with cloud-based computational environments also supports scalability. Containerized workflows or “metabolomics notebooks” can execute end-to-end analyses—from preprocessing to semi-quantification and exposure tiering—with minimal human intervention. Incorporating explainable-AI components further strengthens credibility by revealing which molecular or instrumental features drive a particular abundance prediction, aligning with current demands for interpretable regulatory science.

### 6.4 Closing the loop: from data to decision

Embedding quantification into AI-driven exposomics completes the analytical feedback loop envisioned for the Human Exposome Project. Each new dataset enriches model training, each model refines concentration inference, and each quantitative inference feeds into risk assessment and policy prioritization. This iterative “detect–predict–prioritize–learn” cycle mirrors the adaptive nature of modern toxicology and allows exposure science to keep pace with the ever-expanding chemical universe.

**Figure 4:**
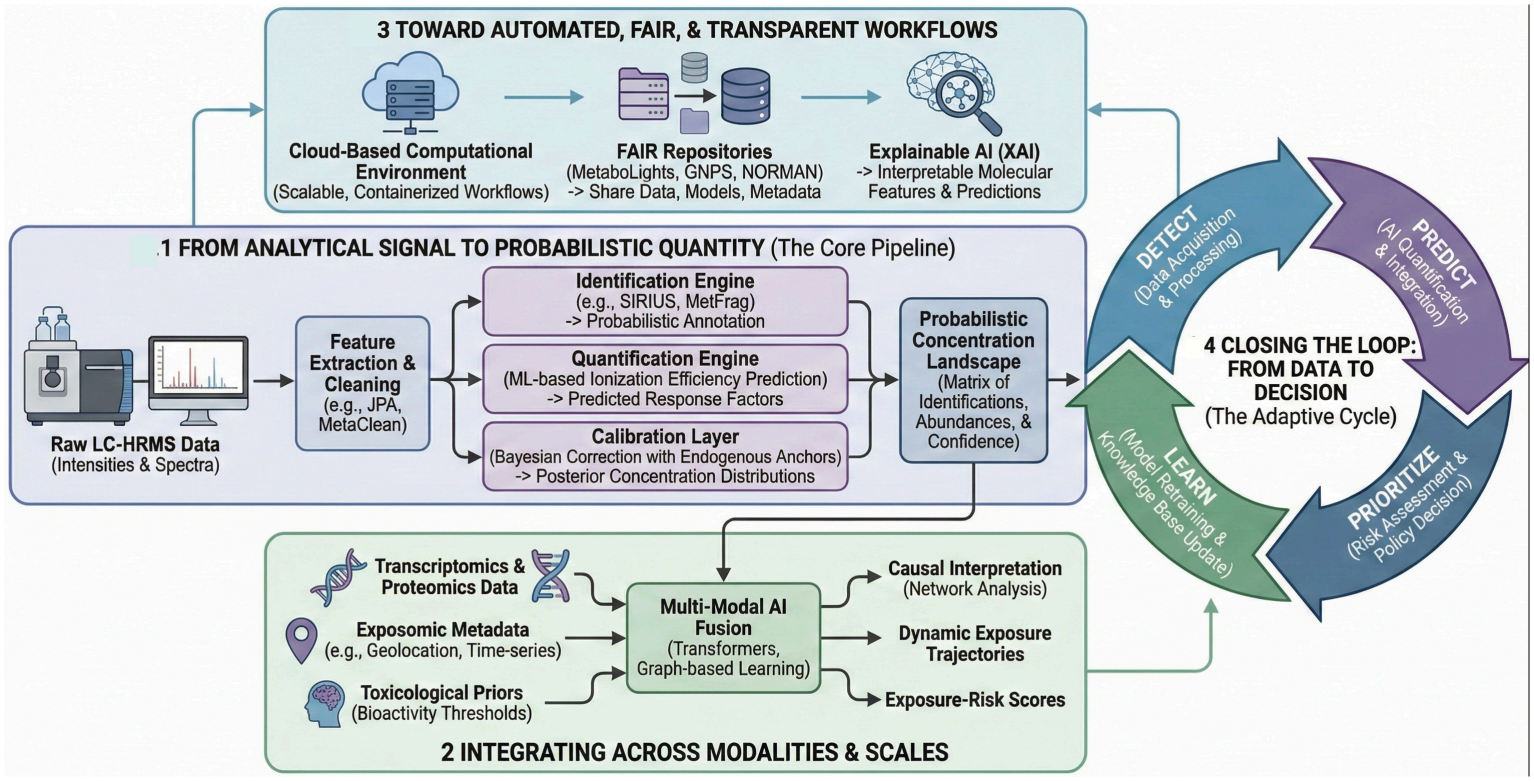
Integrating quantification into AI-driven exposomics pipelines. This diagram illustrates a comprehensive framework for embedding quantitative inference into untargeted metabolomics workflows using artificial intelligence. (6.1) From Analytical Signal to Probabilistic Quantity (The Core Pipeline): Raw LC-HRMS data undergoes feature extraction and cleaning before being processed by parallel identification and quantification engines. The quantification engine uses machine learning to predict ionization efficiency, which is then refined by a Bayesian calibration layer using endogenous metabolite anchors to generate a probabilistic concentration landscape. (6.2) Integrating Across Modalities and Scales: The resulting quantitative data is fused with multi-omics data (transcriptomics, proteomics), exposomic metadata (geolocation, time-series), and toxicological priors through multi-modal AI fusion models (e.g., Transformers) to enable causal interpretation, dynamic trajectory analysis, and exposure-risk scoring. (6.3) Toward Automated, FAIR, and Transparent Workflows: The framework is supported by cloud-based computational environments for scalability, FAIR repositories for sharing data and models, and Explainable AI (xAI) tools to ensure transparency and reproducibility. (6.4) Closing the Loop: From Data to Decision (The Adaptive Cycle): The entire process forms an iterative cycle where data acquisition (Detect) feeds into AI-driven prediction and integration (Predict), informing risk assessment and policy decisions (Prioritize), which subsequently guides model retraining and knowledge base updates (Learn).

In essence, AI makes it possible to treat metabolomics not merely as a sensor of chemical presence but as an intelligent estimator of chemical burden (Figure 4). When integrated with identification workflows, probabilistic calibration, and biological context, quantitative AI pipelines will turn untargeted LC-HRMS into a central engine for evidence-based environmental health decisions. Box 2 provides a preliminary Implementation Checklist for Semi-Quantitative Pipelines.

Noteworthy, tools such as MetaQuant, B-MIS, and the Retip framework allow users to couple ionization-efficiency prediction with metabolite annotation, automating the conversion of LC-HRMS intensities into exposure-relevant quantities. We did not present an end-to-end, dataset-driven demonstration of automated outputs; the pipeline is a conceptual integration requiring empirical benchmarking. The suggested AI-enabled quantification pipeline need still to be designed to support semi-automated estimation of response factors and concentration tiers, contingent on validation and QA/QC.

## 7 Combination of identified and quantified chemicals with predictive toxicology

### 7.1 Bridging exposure and hazard through AI

The workflows described in the previous sections allow for the transition of untargeted metabolomics from a qualitative “fingerprint” to a quantitative inventory of chemical exposures and perturbation of endogenous metabolites. However, the identification and quantification of a molecule are only the initial steps in risk assessment; the critical subsequent step is determining the potential biological impact of these exposures. Historically, toxicology has relied on observational animal studies, but the field is undergoing a transformation into a data-rich discipline—termed “ToxAIcology”—that leverages AI to make sense of big data (35, 36).

Once a chemical feature is identified (Part I) and its concentration estimated (Part II), it serves as the input for AI-driven predictive modeling. The convergence of computational power and massive toxicological datasets now enables ML and deep learning (DL) algorithms to predict hazard endpoints directly from chemical structures, often outperforming traditional animal tests in reproducibility (62). This integration effectively closes the loop between the “exposome” (what we are exposed to) and the “Toxome” (pathways of toxicity) (37, 38), facilitating a move toward high-throughput, mechanistic safety assessment.

### 7.2 From structure to probabilistic hazard prediction

The identification of a metabolite provides the structural information necessary to query predictive models. While early computational toxicology relied on rule-based expert systems and traditional Quantitative Structure-Activity Relationships (QSARs), the current landscape is dominated by Deep Learning (DL) (39). DL models, such as those trained on the Tox21 and ToxCast datasets, can learn complex, non-linear relationships between molecular representations and bioactivity, enabling the prediction of diverse endpoints ranging from mutagenicity to developmental toxicity.

Crucially for exposomics, where many detected features may be novel or data-sparse, modern AI approaches like Read-Across Structure Activity Relationships (RASAR) leverage vast databases to infer toxicity by computing similarities across millions of compounds. Furthermore, emerging techniques in “zero-shot” and “few-shot” learning allow models to generalize to new chemical classes or rare toxicity endpoints without requiring extensive labeled training data (40, 58). This capability is essential for assessing the “chemical dark matter” often revealed in untargeted metabolomics.

### 7.3 Integrating quantity: probabilistic risk assessment and IVIVE

The semi-quantitative concentration estimates derived from AI-calibrated metabolomics (as discussed in Sections 3 and 4) are vital for moving from hazard identification to risk assessment. AI facilitates Probabilistic Risk Assessment (ProbRA) (41–43) by incorporating probability distributions rather than single point estimates, thereby capturing the inherent variability and uncertainty in exposure data.

By combining estimated internal concentrations with *in vitro* bioactivity data, AI tools can perform quantitative *in vitro*-to*-in vivo* extrapolation (IVIVE) (76). Resources such as the Integrated Chemical Environment (ICE) (77) allow for the simulation of toxicokinetics and reverse dosimetry, linking internal metabolite concentrations to external exposure doses and potential adverse outcomes. This allows researchers to contextualize whether the micromolar or nanomolar concentrations detected in a sample exceed safety thresholds, effectively screening for priority hazards among thousands of features.

Box 2 **Practical implementation checklist for semi-quantitative pipelines.**

**Pre-analysis setup:**

□ Compile panel of 20-50 endogenous metabolites with literature concentration ranges
□ Verify metabolite detection across expected concentration range
□ Include pooled QC samples every 10 injections
□ Prepare system suitability standards

**Data acquisition:**

□ Record full MS1 spectra (not just target lists)
□ Maintain consistent injection volume and flow rate
□ Monitor internal standard response throughout run
□ Document any deviations or instrument issues

**Computational processing:**

□ Apply drift correction using pooled QC samples
□ Identify endogenous anchors with >80% confidence
□ Calculate molecular descriptors for all features
□ Train/apply ionization efficiency model
□ Generate concentration tiers with confidence intervals

**Quality assessment:**

□ Verify anchor metabolites span 3+ orders of magnitude
□ Check model cross-validation error (< 10-fold preferred)
□ Flag features outside chemical applicability domain
□ Compare subset against targeted measurements if available

**Reporting:**

□ Document all parameters per Box 1
□ Report tier assignments with confidence levels
□ Deposit raw data in public repository
□ Share model code and calibration data

### 7.4 Causal inference and multimodal integration

To strengthen the biological plausibility of these predictions, the field is moving toward Multimodal AI and Causal AI. Multimodal models can integrate heterogeneous data streams—combining the chemical structure of the identified metabolite, its quantified abundance, and associated multi-omics data (e.g., transcriptomics)—to refine toxicity predictions.

Simultaneously, Causal AI frameworks allow for the reconstruction of AOPs, moving beyond statistical correlation to identify the molecular initiating events driven by specific exposures. By mapping identified metabolites to causal networks, researchers can distinguish between adaptive biomarkers and drivers of toxicity, enhancing the mechanistic relevance of the assessment.

### 7.5 Toward digital twins and automated validation

**Figure 5:**
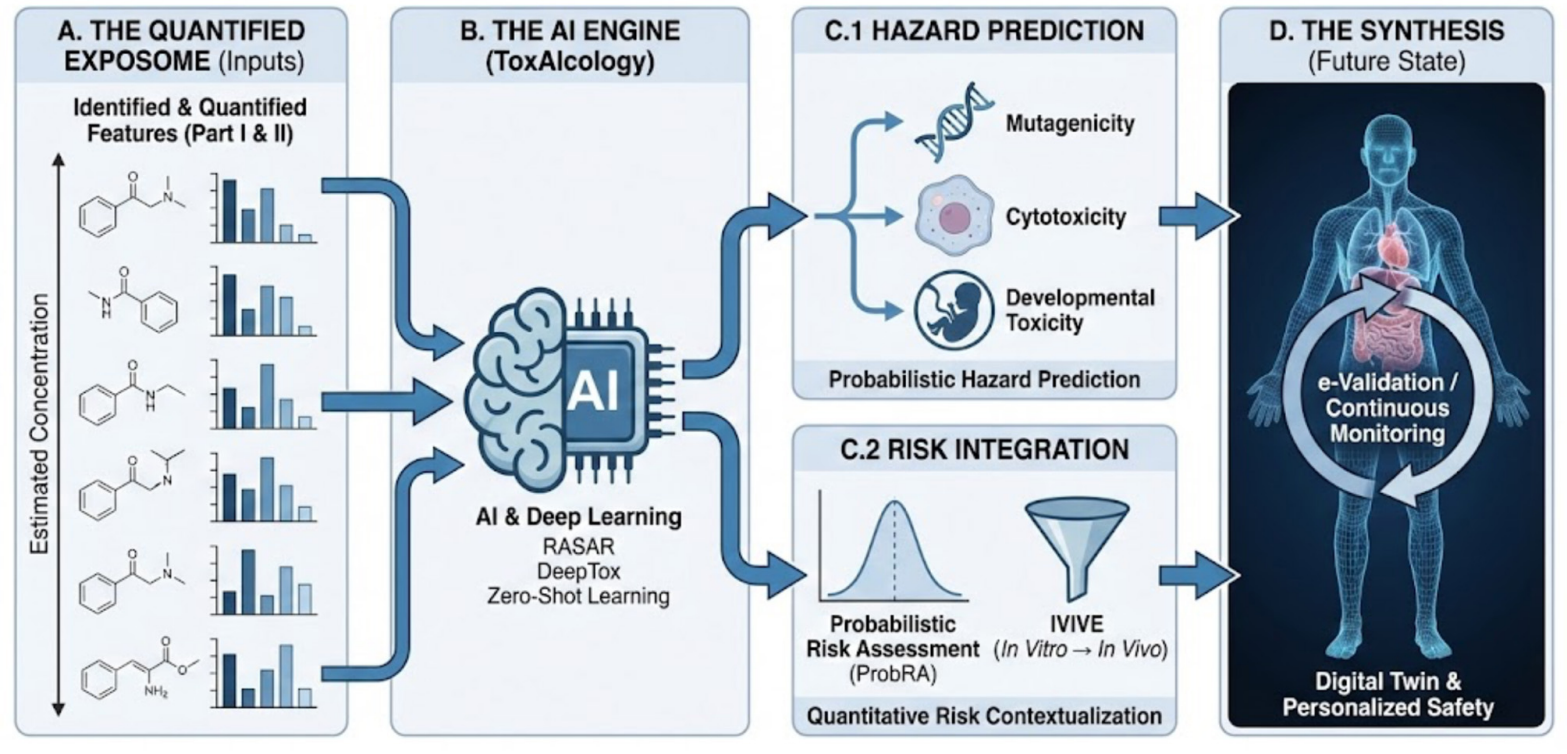
The ToxAIcology convergence: bridging exposure and hazard. This schematic illustrates the integrated workflow described in Section 7, moving from the “Exposome” to the “Toxome.” **(A)** Input: The workflow begins with the output of Parts I and II: a list of identified chemical features with semi-quantitative concentration estimates. **(B)** AI-Driven Prediction: Structural data is fed into Deep Learning (DL) and Read-Across Structure Activity Relationship (RASAR) models to predict hazard endpoints (e.g., mutagenicity, developmental toxicity), utilizing “zero-shot” learning for data-sparse chemicals. **(C)** Risk Integration: Quantitative data is processed through Probabilistic Risk Assessment (ProbRA) and *in vitro*-to-*in vivo* extrapolation (IVIVE) tools (e.g., ICE) to contextualize exposure against safety thresholds. **(D)** Advanced Synthesis: Multimodal and Causal AI integrate multi-omics data to map Adverse Outcome Pathways (AOPs), culminating in the development of “Digital Twins” for personalized susceptibility modeling and “e-validation” for continuous model monitoring.

The ultimate synthesis of quantitative exposomics and predictive toxicology is the creation of “Digital Twins”—computational replicas of individual biological systems (Figure 5). By feeding personalized metabolomic profiles into these AI-driven models, it becomes possible to simulate individual susceptibility and predict health trajectories under specific exposure scenarios.

However, the rapid evolution of these AI models necessitates a dynamic validation paradigm. The concept of “e-validation” (79, 80) proposes using AI to continuously monitor model performance, simulate validation studies, and ensure that predictions remain robust as new chemical spaces are explored. This ensures that the combination of quantified exposure data and predictive toxicology remains trustworthy for regulatory decision-making.

## 8 The threshold of toxicological concern: linking quantification and hazard identification to risk

### 8.1 From concentrations to concern levels

As untargeted metabolomics matures from qualitative detection to quantitative inference, the next logical step is to interpret these concentrations in the context of biological risk. The Threshold of Toxicological Concern (TTC) provides a scientifically grounded benchmark for doing so (8). TTC defines a human exposure level below which adverse health effects are considered negligible, which is particularly useful in the absence of compound-specific toxicity data (44–46, 61). It thus represents a pragmatic bridge between the analytical outputs of exposomics and the decision frameworks of risk assessment.

In essence, semi-quantitative metabolomics provides *how much is there*; TTC contextualizes *whether it matters*. By comparing estimated internal or external concentrations derived from LC–HRMS data to TTC thresholds, exposures can be rapidly stratified into tiers of toxicological relevance. This allows prioritization of the minority of chemicals whose modeled levels approach or exceed TTC cutoffs while confidently classifying the majority as low concern.

### 8.2 Evolution and regulatory acceptance of TTC

The TTC concept has undergone extensive validation and regulatory uptake across diverse domains. It underpins the U.S. FDA's Threshold of Regulation for food contact materials (1995), JECFA's evaluation of flavoring agents, EFSA's guidance for food safety and impurities in pharmaceuticals (ICH M7), and the EU's REACH and cosmetics frameworks (47, 48). Classical TTC thresholds derive from the 5^th^ percentile of NOAEL distributions across thousands of substances, adjusted by a 100-fold uncertainty factor, yielding exposure thresholds of approximately 30, 9, and 1.5 μg/kg/day for Cramer Classes I–III, 0.3 μg/kg/day for organophosphates/carbamates, and 0.0025 μg/kg/day for DNA-reactive genotoxicants

Our 2025 international TTC workshop (Kopanska et al., in preparation)^1^ reaffirmed these values while highlighting emerging refinements. Among the most discussed innovations were endpoint-specific TTCs (e.g., for developmental or neurotoxic effects), internal TTCs (iTTCs) (78) based PBPK modeling, and probabilistic TTCs expressing uncertainty intervals rather than fixed cutoffs. These developments align closely with the goals of quantitative exposomics—moving from deterministic thresholds toward probability distributions of risk.

### 8.3 Integrating TTC with quantitative metabolomics

**Figure 6:**
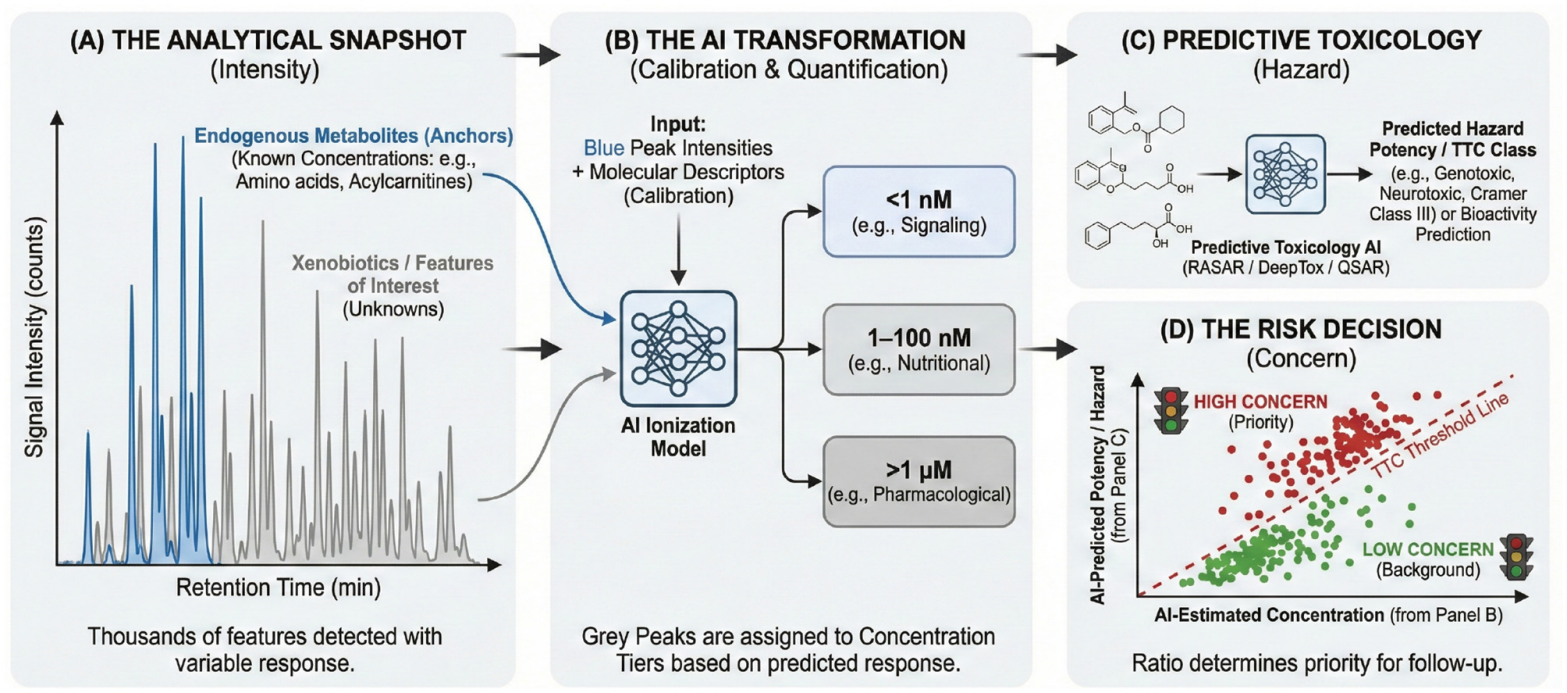
From intensity to quantity to predictive toxicology to concern. This conceptual workflow illustrates the integration of AI-driven quantification and predictive toxicology to operationalize the Human Exposome Project. **(A)** Untargeted LC-HRMS detects thousands of features; a subset of identified endogenous metabolites (e.g., acylcarnitines, amino acids) with known physiological ranges serves as internal “anchors.” **(B)** An AI model, calibrated by these anchors, predicts the ionization efficiency of detected xenobiotics, converting raw signal intensities into semi-quantitative concentration tiers (e.g., nanomolar vs. micromolar). **(C)** Simultaneously, the chemical structures of these features are processed by predictive toxicology models (e.g., RASAR, Deep Learning) to estimate hazard potency or assign Threshold of Toxicological Concern (TTC) classes. **(D)** Finally, the AI-estimated internal concentration is compared against the predicted hazard threshold. This ratio determines the “Level of Concern,” allowing researchers to filter thousands of features down to a prioritized list of biologically relevant exposures.

The semi-quantitative frameworks described earlier (Sections 3–6) generate concentration estimates for thousands of detected compounds, often within one order of magnitude of true abundance. When these estimated values are compared against TTC-derived exposure limits, they can serve as the first *risk calibration* step in an exposome-wide prioritization pipeline (Figure 6).

A practical workflow involves:

6. Estimating internal concentrations from LC–HRMS data using AI-based ionization-efficiency models.
7. Translating to external equivalent doses via toxicokinetic modeling or assumed steady-state conditions.
8. Comparing against TTC thresholds appropriate for structural class or genotoxic potential (e.g., Cramer Class III = 1.5 μg/kg/day, or 0.0025 μg/kg/day for DNA-reactive alerts).
9. Classifying exposures into concern tiers—“below TTC,” “approaching TTC,” or “above TTC”—analogous to probabilistic risk bins.

For example, the LC-HRMS analysis yield signal intensities allowing the analyzed compound to be binned into the 10–100 nM concentration tier. Considering relevant kinetic parameters, the upper and lower concentration ranges can be translated into corresponding daily human exposure doses. These estimated doses can then be compared to TTC-based exposure limits which are predefined for specific chemical classes (e.g., Cramer Class III, 1.5 μg/kg/day for benzene derivatives). Results of such comparisons can guide whether a compound requires further testing or can be considered of low or no concern.

This integration transforms metabolomics from a descriptive inventory into a *decision-ready risk matrix*, allowing rapid triage of detected xenobiotics. The approach is already mirrored in regulatory practice: Health Canada's Chemicals Management Plan recently applied a TTC-based decision tree (81) to 237 substances, enabling roughly one-third to be ruled out as low concern based solely on TTC screening (82).

### 8.4 Toward probabilistic and internal TTCs

The next frontier is harmonizing TTC with *internal* and *probabilistic* frameworks. Internal TTC (iTTC) translates external thresholds into estimated plasma or tissue concentrations using PBPK models. For example, a Cramer Class III external TTC of 1.5 μg/kg/day corresponds roughly to steady-state plasma concentrations in the low micromolar range, assuming conservative kinetics. Such internal thresholds can be directly compared with the micromolar-tier concentration estimates now obtainable through semi-quantitative metabolomics (7).

Probabilistic TTCs extend this concept by expressing the likelihood that a given exposure exceeds the 95% or 99% confidence bound of historical toxicity distributions. In this framework, metabolomics-derived concentrations are not judged against a single deterministic line but are assigned a probability of exceeding TTC—a natural complement to the Bayesian models already applied to quantify uncertainty in ionization-efficiency prediction (Sections 4 and 6). Together, these probabilistic approaches pave the way for quantitative, uncertainty-aware risk triage.

### 8.5 Good TTC Practice and quantitative exposomics

To ensure transparency and reproducibility, the 2025 workshop recommended establishing Good TTC Practice (GTP) analogous to Good Read-Across or Good Quantification Practice. Key elements include:

- Clear documentation of the structural and toxicological domain of applicability;
- Standardized use of up-to-date datasets such as ToxVal (83), ToxRefDB (84), and curated high-throughput screening data;
- Harmonized definitions of exclusion categories (e.g., metals, nanomaterials, potent genotoxins, endocrine-active substances);
- Regular revision of thresholds as new NAMs and probabilistic models emerge;
- Integration of TTC with FAIR data infrastructures for version-controlled application.

Embedding GTP into exposomic workflows will allow the seamless linking of semi-quantitative concentration tiers with probabilistic TTC screening. This combination forms a tiered risk-assessment continuum, where metabolomics provides the quantitative inputs and TTC defines the interpretive thresholds.

### 8.6 TTC in the human exposome project

Within the Human Exposome Project, TTC serves as the organizing principle for connecting exposure measurement to health inference. Quantitative metabolomics supplies an unprecedented resolution of internal chemical concentrations; TTC provides the context for which of those levels merit concern. The integration of TTC thresholds with AI-driven quantification creates a scalable decision framework capable of ranking thousands of exposures by both magnitude and plausibility of harm.

In this vision, the exposome becomes a probabilistic risk landscape: every detected molecule carries an estimated concentration, an uncertainty range, and a TTC-derived probability of exceeding health-relevant concern. This synthesis of quantitative exposure data and toxicological inference moves exposomics beyond discovery science toward evidence-based exposure governance, supporting regulatory strategies that minimize animal testing and focus resources where risk is most credible.

## 9 Current limitations and outlook

**Figure 7:**
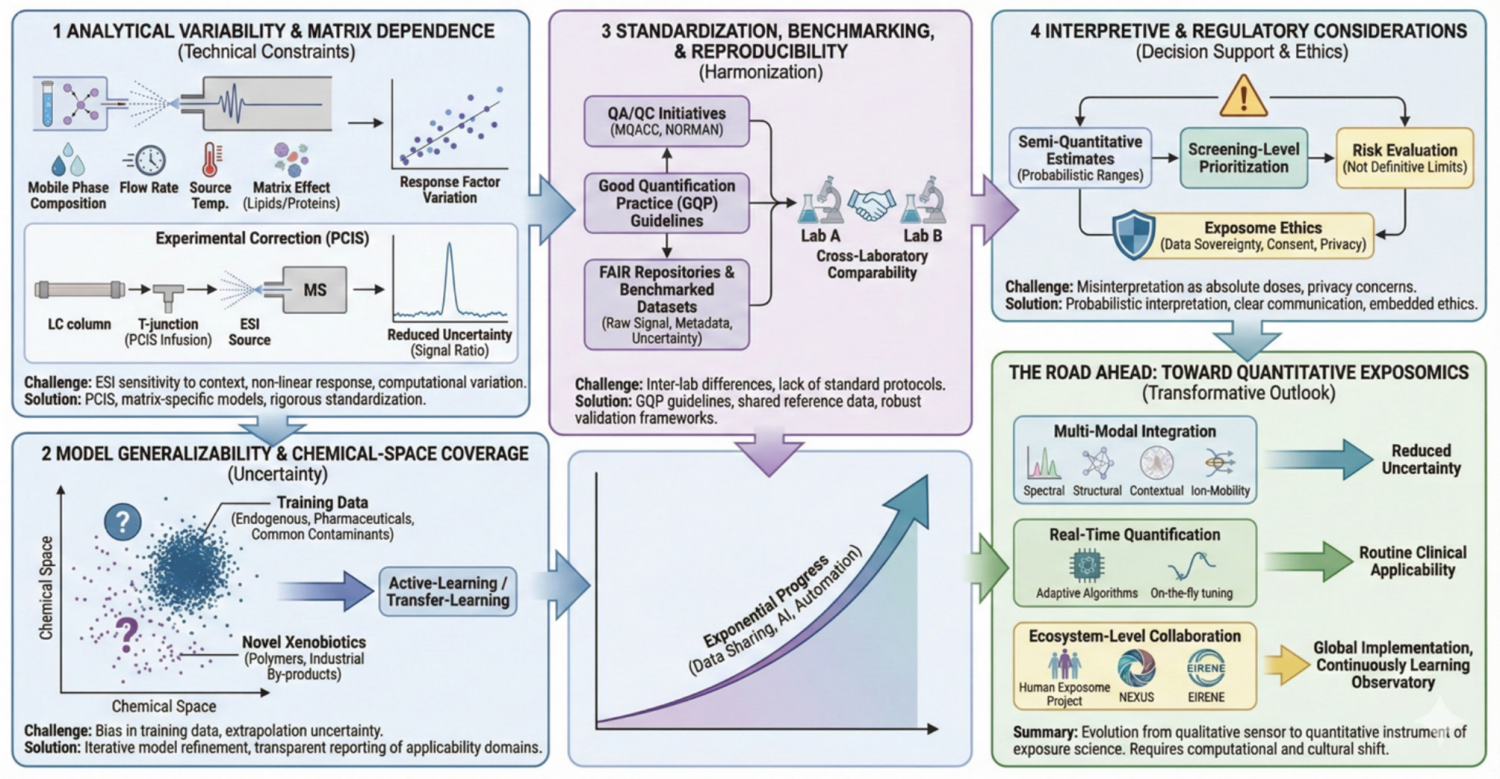
Current limitations and outlook for AI-driven quantitative exposomics. This diagram illustrates the key challenges and future directions for transitioning untargeted metabolomics into a quantitative exposure science. (1) Analytical Variability and Matrix Dependence: Highlights technical constraints, showing how electrospray ionization (ESI) sensitivity to instrumental parameters and matrix effects (e.g., lipids/proteins) leads to response factor variation. Experimental strategies like Post-Column Infusion (PCIS) and matrix-specific models are presented as solutions to reduce uncertainty. (2) Model Generalizability and Chemical-Space Coverage: Addresses the uncertainty arising from bias in training data (endogenous/pharmaceuticals) when extrapolating to novel xenobiotics. Active-learning and transparent reporting of applicability domains are proposed to improve generalizability. (3) Standardization, Benchmarking, and Reproducibility: Emphasizes the need for harmonization across laboratories through QA/QC initiatives (e.g., MQACC, NORMAN), Good Quantification Practice (GQP) guidelines, and FAIR repositories for benchmarked datasets to ensure comparability. (4) Interpretive and Regulatory Considerations: Cautions against misinterpreting semi-quantitative estimates as absolute doses, advocating for their use in probabilistic screening-level prioritization. It also underscores the importance of embedding exposome ethics (data sovereignty, consent, privacy). (The Road Ahead) Toward Quantitative Exposomics: Outlines the transformative outlook driven by exponential progress in data sharing and AI. Future advancements include multi-modal integration, real-time quantification, and ecosystem-level collaboration (e.g., Human Exposome Project, EIRENE), leading to reduced uncertainty, routine clinical applicability, and a global, continuously learning exposome observatory.

Despite rapid progress, the path from qualitative identification to quantitative inference in untargeted metabolomics remains challenging (Figure 7). The integration of AI-based ionization modeling and endogenous calibration represents a conceptual breakthrough, which to the best of our knowledge has not yet been suggested. However, its widespread implementation will require careful attention to analytical, computational, and interpretive limitations. A clear bottleneck is the dependence on knowing the structure, where current automated annotation software are far from perfect (7). Pragmatically, if a feature could represent different structures, the worst case, i.e., the most hazardous substance might be assumed to prioritize actual identification if TTC are exceeded. Recognizing these boundaries is essential to ensure that semi-quantitative data are both credible and actionable for exposomics and public health.

### 9.1 Analytical variability and matrix dependence

A major technical constraint is the context-dependence of ionization efficiency. Electrospray ionization is sensitive to mobile-phase composition, matrix constituents, and instrumental tuning. Even small variations in solvent additives, flow rates, or source temperature can shift response factors by orders of magnitude. This makes cross-platform harmonization difficult and necessitates periodic recalibration or the use of matrix-specific models. Endogenous calibration mitigates some of this variability, but matrix effects—especially in lipid-rich or proteinaceous samples—remain a persistent source of uncertainty, even though such endogenous metabolites are matrix-matched. Furthermore, non-linear detector response can lead to quantitative bias known as “fold-change compression” (49), potentially distorting estimated tiers if not empirically corrected. Beyond instrumental factors, automated data processing itself introduces “computational variation” (50), where algorithm choice and parameter settings can significantly influence quantitative outcomes, necessitating rigorous standardization of computational workflows. Additionally, systematic biases such as “patterned signal ratios” can arise during automated data processing, introducing further computational variation that must be accounted for in quantitative pipelines (50).

Beyond computational response-factor modeling, matrix-effect uncertainty can also be reduced through scalable experimental correction strategies. Postcolumn infusion of standards (PCIS) uses a continuously infused reference compound to track suppression/enhancement across the chromatographic gradient and to correct analyte peak areas via contemporaneous signal ratios (51). In a plasma LC–MS/MS endocannabinoid method, PCIS correction improved matrix-effect estimates, precision, and dilutional linearity for most analytes and enabled parallelization of calibration curves between plasma and neat solution—supporting quantification from neat calibration lines when analyte-free matrix is unavailable (51). Notably, PCIS could outperform stable-isotope internal standards in strong suppression zones, highlighting that even isotopologues may be biased if minor retention shifts cause them to experience different matrix effects (51). For exposomics workflows translating untargeted intensities into concentration estimates, PCIS-derived suppression profiles can therefore serve as an inexpensive QC layer and a potential covariate for AI calibration models, while recognizing that PCIS primarily addresses ionization-stage effects and does not replace controls for extraction recovery or adsorption.

### 9.2 Model generalizability and chemical-space coverage

ML models for ionization efficiency and retention time are inherently limited by the chemical diversity of their training data. Most publicly available datasets are biased toward endogenous metabolites, pharmaceuticals, and common environmental contaminants. Extrapolation to novel xenobiotics—such as polymer additives, industrial by-products, or transformation products—introduces uncertainty that must be explicitly quantified. Active-learning and transfer-learning strategies, where new data iteratively refine the model, will be critical to maintaining predictive accuracy as chemical universes expand. Similarly, transparent reporting of model applicability domains and uncertainty intervals is essential to avoid overinterpretation.

### 9.3 Standardization, benchmarking, and reproducibility

While semi-quantitative models can estimate relative concentrations within 0.5–1 log unit accuracy, results are only meaningful when accompanied by standardized quality-control (QC) frameworks (52). Current metabolomics QA/QC initiatives—such as the Metabolomics Quality Assurance and Quality Control Consortium (MQACC) (53) and the NORMAN non-target screening network—provide templates for harmonizing calibration, validation, and reporting practices. As recently reviewed, establishing such robust quality assurance is a prerequisite for the acceptance of metabolomics in regulatory decision-making (9). Expanding these to include Good Quantification Practice (GQP) guidelines, analogous to Good Cell Culture (86) or Good Read-Across (87) Practices, would support consistency across laboratories. Benchmarked reference datasets (54), shared via FAIR repositories with raw signal, calibration metadata, and uncertainty measures, will be vital for cross-comparison and regulatory trust.

Concrete harmonization recommendations already exist in the exposomics literature. For HRMS-based internal exposome profiling, proposed validation moves beyond qualitative “feature counts” toward quantitative performance characteristics (e.g., recovery, detection/quantification limits, repeatability/reproducibility) evaluated using agreed sets of endogenous and exogenous chemicals and, critically, common reference materials; validation is also recommended for bioinformatics steps (preprocessing/peak-picking) because computational choices can drive variability (10, 85). Complementarily, in the human biomonitoring context, suspect/NTS has been framed as an early-warning approach for chemicals of emerging concern, but large-scale implementation is still limited by harmonization gaps and the need for consolidated reference libraries and multidisciplinary computational capacity (11).

### 9.4 Interpretive and regulatory considerations

Even when approximate concentrations are obtained, translating them into health-relevant conclusions requires caution. Semi-quantitative estimates must be interpreted probabilistically, not as absolute doses. For regulatory application, such data can support screening-level prioritization—identifying compounds that merit targeted quantification or risk evaluation—but not definitive exposure limits. Communicating these distinctions clearly to policymakers and the public will be essential to preserve confidence in exposomics as a decision-support tool. Importantly, as quantitative exposomics becomes more predictive, questions of data ethics and privacy will arise: reconstructed exposure profiles could inadvertently reveal lifestyle, medication, or occupational information. Embedding Exposome Ethics (55)—including data sovereignty, consent, and equitable access—into quantitative pipelines is therefore imperative.

### 9.5 The road ahead: toward quantitative exposomics

The outlook is nonetheless transformative. Advances in multi-modal learning, combining spectral, structural, and contextual data, promise to reduce uncertainty and extend generalizability. Integration of ion-mobility spectrometry, isotopic pattern modeling, and chromatographic metadata into unified AI architectures will yield richer predictive representations of molecular behavior. Real-time quantification during acquisition—driven by adaptive algorithms that adjust ionization parameters on the fly—is an emerging frontier that could bring exposure quantitation closer to routine clinical applicability.

A concrete demonstration of how these concepts translate into deployable semi-quantification comes from food-monitoring workflows developed for untargeted LC–HRMS. In her PhD thesis on semi-quantitative screening of cereals and honey, Wang synthesized response-factor/ionization-efficiency modeling into an end-to-end monitoring pipeline and highlighted a key practical constraint for exposomics: absolute ionization-efficiency scales are instrument-dependent, but relative ionization efficiencies can be stabilized by anchoring to a reference compound and by using “transformation compounds” to harmonize responses across platforms, yielding strong inter-laboratory agreement (reported R^2^ ≈ 0.85) (56). Importantly, the thesis provides realistic error expectations in complex matrices (e.g., average ~2–4-fold error with most compounds within 5–10-fold), helping define the performance envelope within which semi-quantitative exposomics can credibly support screening-level prioritization and downstream risk-oriented interpretation.

At the ecosystem level, collaborative initiatives like the Global Exposome Forum (88), the NEXUS Network for Exposomics in the United States (90), and the European EIRENE infrastructure (89) are creating the data volume and standardization momentum required for global implementation. As semi-quantitative pipelines mature, they will enable a continuously learning exposome observatory—an iterative system where each analyzed dataset refines both chemical knowledge and model accuracy.

In summary, the quantification frontier of metabolomics mirrors the trajectory once faced by genomics: an early phase of technical uncertainty giving way to exponential progress through data sharing, automation, and AI integration. The challenge ahead is not merely computational but cultural—embedding reproducibility, transparency, and ethical foresight into every stage of analysis. As these foundations solidify, untargeted metabolomics will evolve from a qualitative sensor of chemical diversity into a quantitative instrument of exposure science, capable of mapping the chemical dimensions of human health with unprecedented resolution.

## 10 Conclusion

The evolution of metabolomics from a discovery-driven to a quantitatively informed discipline marks a decisive step toward realizing the Human Exposome Project. In the first part of this series, we described how AI enables more confident identification of unknown metabolites through retention-time prediction and multi-parameter annotation. In this second ML learning, probabilistic calibration, and endogenous reference scaling—can transform signal intensity into estimated quantity. Together, these innovations redefine the analytical backbone of exposomics: moving from *what* is detected to *how much* is present, and ultimately, *what it means* for human health.

Quantification remains the critical link between chemistry and biology. Only by approximating internal concentrations can metabolomics bridge the gap between detection and effect, anchoring molecular features to toxicological thresholds, dose—response relationships, and population-level exposure estimates. Through ML models of ionization efficiency, endogenous metabolite calibration, and tiered concentration estimation, we now possess the conceptual tools to approach this goal—even in the absence of authentic standards. While the resulting values are not yet absolute, they provide actionable tiers that contextualize exposures relative to biological activity levels, guiding prioritization for risk assessment and mechanistic follow-up. The integration of experimental design principles, such as those demonstrated by Chu et al. (26), will be critical to move from chemical heuristics to calibrated, reference-free quantification pipelines. Consistent with the “intensity → quantity → concern” framing, Palm and Kruve (13) argue that uncertainty in reference-free concentration estimates (often within an order of magnitude) should be interpreted alongside uncertainty in hazard metrics: reported toxicity endpoints for the same chemical/species commonly vary within ~10-fold (and sometimes much more), and QSAR-based toxicity predictions can show similar spread. In this sense, semi-quantitative exposure estimates that carry explicit uncertainty can still be fit-for-purpose for prioritization in exposome-scale screening, provided uncertainty is propagated into downstream risk-ranking rather than treated as exact concentration.

The broader significance of these developments extends beyond analytical chemistry. Quantitative exposomics establishes the measurement infrastructure for preventive public health, enabling real-world exposure monitoring at population scale. It also fosters a new scientific logic: data-driven, continuously learning, and deeply integrative. Each dataset contributes not only new molecular information but also improved calibration for future studies, creating a feedback loop between experimentation, computation, and policy. In this way, the exposome becomes a living observatory of environmental influences—dynamic, transparent, and globally connected.

The next frontier lies in embedding semi-quantitative metabolomics within AI-driven, FAIR-compliant infrastructures capable of unifying multi-omics and contextual data into coherent exposure narratives. As models grow in precision and coverage, the field will transition from semi-quantitative estimation toward probabilistic quantification—expressing not only what we know but also how certain we are. Ethical governance and data transparency will be essential companions to this progress, ensuring that the growing power of quantitative exposomics serves collective benefit.

Ultimately, the integration of identification and quantification, guided by AI, transforms untargeted metabolomics into a true *measurement science* of the human environment. By quantifying the invisible chemistry that surrounds and shapes us, we move one step closer to the vision articulated at the Exposome Moonshot Forum: an evidence-based, globally coordinated effort to chart the environmental determinants of health. In this synthesis of technology, computation, and public purpose, the exposome becomes not merely a scientific ambition but a cornerstone of 21^st^-century preventive medicine.

## Data Availability

The original contributions presented in the study are included in the article/supplementary material, further inquiries can be directed to the corresponding author.

## Footnotes

1 Kopanska K, Rovida C, Antignac E, Barton-Maclaren T, Corgreave I, Dorne J-L, et al. *From Thresholds to Odds: The Role of TTC in Modern Risk Assessment*. ALTEX Workshop Report (in preparation).
